# *vacA s1m1* genotype and *cagA* EPIYA-ABC pattern are predominant among *Helicobacter pylori* strains isolated from Mexican patients with chronic gastritis

**DOI:** 10.1099/jmm.0.000660

**Published:** 2018-01-10

**Authors:** Josefina Atrisco-Morales, Verónica I. Martínez-Santos, Adolfo Román-Román, Judit Alarcón-Millán, José De Sampedro-Reyes, Iván Cruz-del Carmen, Dinorah N. Martínez-Carrillo, Gloria Fernández-Tilapa

**Affiliations:** ^1^​ Laboratorio de Investigación Clínica, Facultad de Ciencias Químico-Biológicas, Universidad Autónoma de Guerrero, Av. Lázaro Cárdenas s/n C.U. Sur. Chilpancingo, Guerrero, C.P. 39090, Mexico; ^2^​ CONACYT Research Fellow- Universidad Autónoma de Guerrero, Facultad de Ciencias Químico-Biológicas, Universidad Autónoma de Guerrero, Guerrero, Mexico; ^3^​ Laboratorio de Investigación en Bacteriología, Facultad de Ciencias Químico-Biológicas, Universidad Autónoma de Guerrero, Guerrero, Mexico; ^4^​ Hospital General “Dr. Raymundo Abarca Alarcón”, Chilpancingo, Guerrero, Mexico

**Keywords:** *H. pylori*, VacA, BabA, CagA, EPIYA motifs, chronic gastritis

## Abstract

**Purpose:**

Virulent genotypes of *Helicobacter pylori vacA s1m1/cagA^+^/babA2^+^* have been associated with severe gastric diseases. VacA, CagA and BabA are polymorphic proteins, and their association with the disease is allele-dependent. The aims of this work were: (i) to determine the prevalence of *H. pylori* by type of chronic gastritis; (ii) to describe the frequency of *cagA*, *babA2* and *vacA* genotypes in strains from patients with different types of chronic gastritis; (iii) to characterize the variable region of *cagA* alleles.

**Methodology:**

A total of 164 patients with chronic gastritis were studied. Altogether, 50 *H. pylori* strains were isolated, and the status of *cagA*, *babA2* and *vacA* genotypes was examined by PCR. *cagA* EPIYA segment identification was performed using PCR and sequencing of *cagA* fragments of six randomly selected strains.

**Results/Key findings:**

The overall prevalence of *H. pylori* was 30.5 %. Eighty percent of the isolated strains were *vacA s1m1*, and the *cagA* and *babA2* genes were detected in 74 and 32 % of the strains, respectively. The most frequent genotypes were *vacA s1m1/cagA^+^/babA2^-^* and v*acA s1m1/cagA^+^/babA2*^+^, with 40 % (20/50) and 28 % (14/50), respectively. In *cagA*^+^, the most frequent EPIYA motif was -ABC (78.4 %), and EPIYA-ABCC and -ABCCC motifs were found in 10.8 % of the strains. A modified EPIYT-B motif was found in 66.6 % of the sequenced strains.

**Conclusion:**

*H. pylori* strains carrying *vacA s1m1*, *cagA*^+^ and *babA2*^-^ genotypes were the most prevalent in patients with chronic gastritis from the south of Mexico. In the *cagA*^+^ strains, the EPIYA-ABC motif was the most common.

## Introduction

*Helicobacter pylori* is a Gram-negative bacterium that colonizes the gastrointestinal tract of humans, mainly the gastric mucosa. These bacteria colonize around half of the world's population, although their prevalence varies among geographical regions within a country, as well as between rural and urban areas, due to the socio-economic conditions, age and population ethnicity [1, 2]. Even though 80 % of the infected population is asymptomatic, persistent infection by these bacteria causes chronic inflammation of the mucosa, which manifests as gastritis that can develop to chronic atrophic gastritis, intestinal metaplasia, dysplasia, and finally, gastric cancer [3–5]. Prevalence of infection by *H. pylori* and distribution of virulent strains, together with host factors, determine the regional variations in the incidence of gastric diseases [6].

Among *H. pylori* strains associated with severe gastric diseases are those that carry the *babA2* and *cagA* genes, especially in combination with the genotype of *vacA*, *s1m1* [7]. *vacA* encodes the protein VacA, a vacuolating cytotoxin secreted through the type V secretion system (T5SS) or autotransporter [8]. Besides forming pores in the cell membrane, this protein also induces apoptosis and inhibits cell proliferation and effector T-cell functions [9]. The *vacA* gene is present in all *H. pylori* strains. It has several isoforms, of which *s1m1* is the most virulent, while the isoform *s2m2* is the least virulent, and combinations of these result in isoforms of intermediate virulence [10]. CagA is an effector protein encoded in the pathogenicity island *cag*PAI. This protein is translocated through a type IV secretion system (T4SS) encoded in the same PAI, to the cytoplasm of the gastric cells where it is phosphorylated by Src and Ab1 kinases [11–13]. It is recognized as an oncoprotein because its phosphorylated form has affinity for proteins with phosphorylated tyrosine-binding domains, like tyrosine phosphatase Shp2. The interaction between CagA and its partners leads to the activation of signalling cascades involved in cell proliferation, apoptosis, cell cycle suppression and inflammatory responses in both phosphorylation-dependent and -independent manners [14]. The phosphorylation site of CagA is located on its C-terminal region, and comprises tyrosine residues that are part of a 5-amino acid sequence known as the EPIYA (Glu-Pro- Ile-Tyr-Ala) motif [12]. Four EPIYA motifs have been identified so far: EPIYA-A, -B, -C and -D, which are distinguished by the amino acid sequences that flank them. *H. pylori* strains that carry EPIYA-ABC motifs are found in Western countries, while those that carry EPIYA-ABD motifs are characteristic of Asian countries [15]. Western strains can produce variants of the CagA cytotoxin with up to five EPIYA-C motifs. Phosphorylation of CagA occurs mainly on the tyrosine residues of EPIYA-C and -D motifs, and the level of phosphorylation, as well as the carcinogenic potential, are related to a higher number of EPIYA-C motifs or to the presence of EPIYA-D [15–18]. Lastly, BabA is an afimbrial adhesin that binds Lewis^b^ antigens in the gastric mucosa, thus inducing an autoimmune response to Lewis antigens, facilitating colonization, and increasing the response of IL-8 [19–21]. Its interaction with its receptor enhances CagA translocation, thus favouring the inflammatory response [22]. The *babA* gene has two isoforms *babA*1 and *babA*2, of which, *babA*2 encodes a functional protein [21].

The aims of this work were: (i) to determine the prevalence of *H. pylori* by type of chronic gastritis; (ii) to determine the frequency of *cagA*, *babA2* and *vacA* genotypes in *H. pylori* strains isolated from Mexican patients diagnosed with different types of chronic gastritis; and (iii) to characterize the variable region of *cagA* alleles that encode the C-terminal region of CagA in order to determine the type and number of EPIYA motifs, as well as the frequency of their combinations.

## Methods

### Patients

A cross sectional study was performed with 164 patients (61.6 % female, 38.4 % male) that were attended to at the Gastroenterology Service at the General Hospital ‘Dr Raymundo Abarca Alarcón’ and Specialized Unit in Gastroenterology Endoscopy, in Chilpancingo, Guerrero, Mexico. Patients were sequentially selected among those who attended for an endoscopic study due to dyspepsia symptoms. Only patients that had no *H. pylori* eradication treatment one month prior to the endoscopic procedure were selected. None of the patients included in this study were under treatment with proton pump inhibitors or with gastric pH neutralizing agents within 15 days prior to biopsy. Patients receiving non-steroidal anti-inflammatory therapy were excluded from the study. All patients signed a letter of consent. This project was approved by the Bioethics Committee of the Autonomous University of Guerrero, by the Department of Education and Research of the General Hospital ‘Dr Raymundo Abarca Alarcón’, and by the authorized personnel of the Specialized Unit in Gastroenterology Endoscopy.

### Biopsies

The endoscopic study was performed after a fasting night with a video processor and video gastroscope (Fujinon, Wayne, NJ, USA). Two biopsies were taken from the antrum, one was immediately fixed in 10 % formalin for histological examination, and the other one was placed in Brain Heart Infusion Broth (BHI) (Becton Dickinson, NC, USA) with 10 % glycerol for the isolation of *H. pylori*. The biopsies were transported at 4 °C, and those intended for isolation of *H. pylori* were processed immediately.

### Histology

Formalin-fixed biopsies were embedded in paraffin. Tissue sections of 4 µm were stained with hematoxylin-eosin for histological study. The histopathological diagnosis was carried out according to the updated Sydney system [23]. Endoscopic and histopathological findings were only used to diagnose patients.

### Isolation and identification of *H. pylori*

Each biopsy transported in BHI broth with 10 % glycerol was macerated with a sterile wood applicator. In total, 50 µl of the homogenates were cultivated on Columbia Agar plates (Becton Dickinson, NC, USA) added with 10 % ram blood, IsoVitaleX Enrichment and *Helicobacter pylori* selective supplement Dent (10 mg/L of vancomycin, 5 mg/L of trimethroprim, 5 mg/L of cefsulodin, 5 mg/L of amphotericin B) (Oxoid, Basingstoke, UK) at pH 6.8–7.0. The homogenates were distributed on the culture medium by isolation strip. The inoculated plates were incubated under microaerophilic conditions with 5 % O_2_, and 5 % CO_2_ at 37 °C in GasPak jars for 3–7 days. *H. pylori* was identified by colony morphology (small, transparent colonies, 1 mm in diameter), Gram staining and biochemical tests (urease, catalase and oxidase positive). *H. pylori* strain ATCC 43504 was used as a positive control.

### DNA purification

Isolates identified as *H. pylori* were subcultured and incubated for 72 h. A pool of colonies from each biopsy was resuspended in extraction solution (10 mM Tris pH 8, 10 mM EDTA, 0.5 % SDS) for digestion with proteinase K. Total DNA was obtained by the phenol: chloroform: isoamylic alcohol technique [24]. Total DNA concentration was determined in a NanoDrop 2000 (NanoDrop Technologies, Wilmington, DE, USA). All DNA samples were stored at −20 °C until use.

### Molecular confirmation and genotypification of *vacA, cagA* and *babA2* of *H. pylori* strains

Confirmation of *H. pylori* strains was performed using oligonucleotides 16S1 and 16S2 (Table 1), which amplify a 522 bp fragment of the *16S* rRNA, according to the method described by Román-Román *et al*. [25]. *vacA* genotyping and the status of *cagA* and *babA2* were assessed by PCR with oligonucleotides specific for each region and gene (Table 1). The reaction mixture contained 1.5 mM MgCl_2_; 0.2 mM dNTPs; 2.5 pmol of oligonucleotides F1 and B1, or 5 pmol of VAGF and VAGR, or 2.5 pmol of VAIF and VAIR, or 12.5 pmol of babA2F and babA2R; 1.5 U of Taq DNA polymerase (Invitrogen, Carlsbad, CA, USA) and 200 ng of DNA, in a total volume of 25 µl. Amplification conditions were: one cycle at 94 °C for 10 min; 35 cycles at 94 °C for 1 min, 57 °C for 1 min, 72 °C for 1 min; and a final extension cycle at 72 °C for 10 min. The PCR products were subjected to 2.5 % agarose gel electrophoresis, stained with ethidium bromide and visualized with ultraviolet light (UV). In each PCR, DNA from strain ATCC 43504 (*vacAs1m1*/*cagA*^+^/*babA2*^+^) was used as a positive control, and as a negative control, DNA was replaced with sterile deionized water. All reactions were performed in a Mastercycler Ep gradient thermal cycler (Eppendorf, Hamburg, Germany).

**Table 1.** Oligonucleotides used in this work

**Gene**	**Oligonucleotide**	**Sequence**	**Size (bp)**	**Reference**
*16S rRNA*	16S1	5′-GCTAAGAGATCAGCCTATGTCC-3′	522	[50]
	16S2	5′-CAATCAGCGTCAGTAATGTTC-3′		
*vacAs1, vacAs2*	VAIF	5′-ATGGAAATACAACAAACACAC-3′	259, 286	[10]
	VAIR	5′-CTGCTTGAATGCGCCAAAC-3′		
*vacAm1, vacAm2*	VAGF	5′-CAATCTGTCCAATCAAGCGAG-3′	570, 645	[51]
	VAGR	5′-GCGTCTAAATAATTCCAAGG-3′		
*cagA*	F1	5′-GATAACAGGCAAGCTTTTGAGG-3′	349	[45]
	B1	5′-CTGCAAAAGATTGTTTGGCAGA-3′		
*babA2*	bab2F	5′-AATCCAAAAAGGAGAAAAAACATGAAA-3′	850	[20]
	bab2R	5′-TGTTAGTGATTTCGGTGTAGGACA-3′		
EPIYA	cagA28F	5′-TTCTCAAAGGAGCAATTGGC-3′		[52]
-A	cagA-P1C	5′-GTCCTGCTTTCTTTTTATTAACTTTAGC-3′	264	
-B	cagA-P2TA	5′-TTTAGCAACTTGAGTATAAATGGG-3′	306	
-C	CagAWest	5′-TTTCAAAGGGAAAGGTCCGCC-3′	501	
-D	CagAEast	5′-AGAGGGAAGCCTGCTTGATT-3′	495	
Empty site	ESf	5′-ACATTTTGGCTAAATAAACGCTG-3′	360	[27]
	ESr	5′-TCATGCGAGCGGCGATGTG-3′		

### Confirmation of *cagA* negative strains

The absence of *cagA* and the pathogenicity island *cag*PAI in the *cagA*^-^ strains was confirmed by the empty-site assay by conventional PCR, using the ESf and ESr oligonucleotides (Table 1), which bind upstream and downstream, respectively, of the region where the *cag*PAI is inserted in the genome of the reference strain NCTC 12455 (NCTC: National Collection of Type Culture) [26]. The PCR mixture contained 50 ng of DNA, 0.08 mM dNTPs (Invitrogen, Carlsbad, CA, USA), 1 mM MgCl_2_, 5 pmol of each oligonucleotide and 1 U of Platinium Taq DNA Polymerase (Invitrogen, Carlsbad, CA, USA), in a final volume of 15 µl. The amplification conditions were: one cycle at 94 °C for 5 min; 35 cycles at 94 °C for 30 s, 61 °C for 30 s and 72 °C for 45 s; and one final extension cycle at 72 °C for 7 min. PCR products were subjected to 2 % agarose gel electrophoresis, followed by ethidium bromide staining and UV light observation. As a negative control, DNA was replaced with sterile deionized water. DNA from strain ATCC43504 (*cagA*^+^, *cag*PAI^+^) was used as a second negative control, and strain UEGE-644 (*cagA*^-^, *cag*PAI^-^) as a positive control. The presence of a 360 bp product was considered indicative of the absence of *cagA* and *cag*PAI [26, 27].

### Detection of *H. pylori* CagA EPIYA motifs

Detection of CagA EPIYA motifs was performed by PCR with DNA from strains previously identified as *cagA*^+^. Four PCR reactions were performed per strain using antisense oligonucleotides cagA-P1C (EPIYA-A), cagA-P2TA (EPIYA-B), cagAWest (EPIYA-C) and cagAEast (EPIYA-D), and the cagA28F sense oligonucleotide (Table 1). Each reaction was carried out using 300 ng of DNA, following the conditions previously described [28]. As a positive control, DNA from strain ATCC 43504 (carrying EPIYA-ABCCC motif) was used, and as a negative control, the DNA was replaced with sterile deionized water.

### Statistical analysis

The statistical program STATA v.12 was used for data analysis. Simple and relative frequencies of the qualitative variables were calculated, and Fisher’s exact test, *X*^2^ test or Student's *t*-test were used to determine differences between groups. A *P-*value <0.05 was considered significant.

## Results

### Patients

**Fig. 1.**
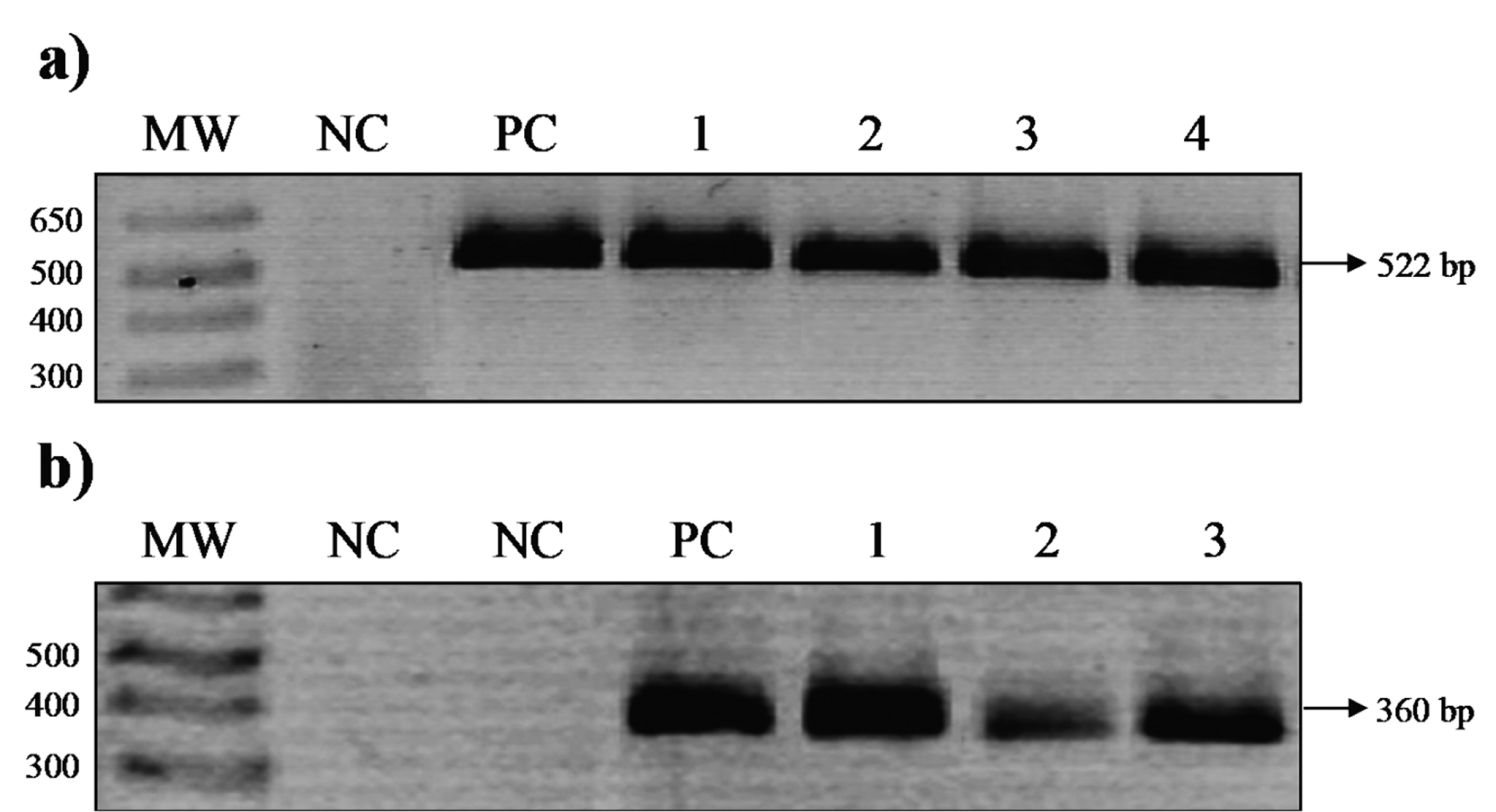
Agarose gel electrophoresis of PCR products. Representative gel electrophoresis of PCR products of (a) *16S* rRNA and (b) *cag*PAI empty site. (a) Lanes: MW, 100 bp molecular weight marker; NC, negative control; PC, positive control (*H. pylori* strain ATCC43504); 1-4, isolated *H. pylori* strains. (b) Lanes: MW, 100 bp molecular weight marker; NC, negative controls (second lane: *H. pylori* strain ATCC43504 *vacAs1m1*/*cagA*^+^/*babA2*^+^); PC, positive control (strain UEGE844 *vacAs2m2*/*cagA*^-^/*babA2*^-^); 1-3, isolated *H. pylori cagA*^-^ strains.

We analysed 164 patients with histopathological diagnosis of chronic gastritis, of which 89.6 % (147/164) showed *H. pylori*-associated chronic gastritis. Of these, 51 % (75/147) had chronic superficial gastritis, 25.2 % (37/147) had active chronic gastritis, and 23.8 % (35/147) had follicular chronic gastritis, while the rest of the patients (10.4 %, 17/147) showed reactive gastritis (Table 2). The mean age of the patients was 48±17 years old (ranging between 19–89 years). In total, 61.6 % (101/164) were female patients, and 34.8 % (57/164) had undergraduate or higher education. The frequency of *H. pylori* isolation was 30.5 % (50/164). No significant differences were found between age, gender or schooling level of *H. pylori* positive and negative patients (Table 2). All strains identified as *H. pylori* by culture were confirmed by amplification of a fragment of the *16S* rRNA gene by PCR (Fig. 1a). Regarding the types of gastritis, 60 % (21/35) of the patients with chronic follicular gastritis, and 35.3 % (6/17) with reactive gastritis were *H. pylori* positive, while those with chronic active gastritis and chronic superficial gastritis showed a lower infection frequency, 24.3 % (9/37) and 18.7 % (14/75), respectively. The frequency of infection was significantly different among diagnoses (*P*<0.001).

**Table 2.** Socio-demographic characteristics, *H. pylori* infection and histopathological diagnosis of 164 patients with chronic gastritis

	**Chronic gastritis**		
	***H. pylori* negative**	***H. pylori* positive**	***P-*value**
	*n*=114 (100 %)	*n*=50 (100 %)	
Age (years; mean±sd)	49±16	48±18	0.609*
Gender, *n* (%)			
Female	75 (65.8)	26 (52)	0.117†
Male	39 (34.2)	24 (48)	
Schooling, *n* (%)			
Unschooled	9 (7.9)	3 (6)	0.190‡
Elementary	28 (24.5)	13 (26)	
Junior high	14 (12.3)	13 (26)	
High school	22 (19.3)	5 (10)	
Undergrad or higher	41 (36)	16 (32)	
Types of chronic gastritis, *n* (%)			
Superficial chronic gastritis	61 (53.5)	14 (28)	<0.001
Active chronic gastritis	28 (24.6)	9 (18)	
Follicular chronic gastritis	14 (12.3)	21 (42)	
Reactive gastritis	11 (9.6)	6 (12)	

*Student’s *t*-test.

†X^2^ test.

‡Fisher’s exact test.

### Genotyping of *vacA, cagA* and *babA2* status

In order to determine the *vacA* genotype and the status of *cagA* and *babA2*, DNA of all isolates was subjected to PCR. Overall, 80% (40/50) of the isolates harboured the *s1m1* allelic variant of *vacA*; 74 % (37/50) carried *cagA* and 32 % (16/50) were positive for *babA2* (Table 3). The most frequent genotypes among the strains were *vacA s1m1/cagA^+^/babA2^-^* and *vacA s1m1/cagA^+^/babA2*^+^, with 40 % (20/50) and 28 % (14/50), respectively. Altogether, 70% (35/50) of the isolates were *vacA s1m1*/*cagA*^+^, and of these, 40 % (14/35) were *babA2*^+^. Of all the patients analysed, only one showed mixed infection with two strains carrying different *vacA* genotypes (*s1m1* and *s1m2*), both *cagA*^+^/*babA2*^-^. Of the strains with *vacA s1m1*/*cagA*^+^ genotype, 54.3 % (19/35) was isolated from female patients. No significant difference was found between *H. pylori* genotype and patient gender.

**Table 3.** Distribution of *vacA*/*cagA*/*babA2* genotypes of *H. pylori* strains per gender of patients with chronic gastritis

**Genotypes**	**Gender**		***P-*value**
	**Female**	**Male**	
	*n*=26	*n*=24	
	***n* (%)**	***n* (%)**	
*vacA s1m1/cagA^+^/babA2^+^*	6 (23)	8 (33)	0.481*
*vacA s1m1/cagA^+^/babA2^-^*	12 (43)	8 (33)	
*vacA s1m1/cagA^-^/babA2^-^*	1 (4)	3 (13)	
*vacA s1m1/cagA^-^/babA2^+^*	1 (4)	0	
*vacA s2m2/cagA^-^/babA2^-^*	5 (19)	3 (13)	
*vacA s1m2/cagA^+^/babA2^-^*	0	1 (4)	
*vacA s1m1* and *s1m2/cagA^+^/babA2^-^*	1 (4)	0	
*vacA s2m2/cagA^+^/babA2^+^*	0	1 (4)	

*Fisher’s exact test.

However, when we analysed the frequency of *H. pylori* according to the types of chronic gastritis, we found that there was a significant difference (Table 4), the absence of the bacterium being more prevalent, except in follicular chronic gastritis where 60 % (21/35) of the cases were *H. pylori* positive. Regarding the genotypes, *vacA s1m1* isolates were the most frequent in all types of gastritis, including reactive gastritis (83 %, 5/6), while *cagA* positive strains predominated in all groups, with frequencies that varied between 55.6 and 85.7 %. The highest frequency of isolates was *babA2* negative in all types of gastritis, and the highest percentage of strains *babA2* positive were isolated from patients with follicular chronic gastritis (35.7 %, 5/14).

**Table 4.** Prevalence of *H. pylori* and *vacA, cagA* and *babA2* genotypes by type of chronic gastritis

	**Types of chronic gastritis**				
	***H. pylori*-associated**			**Non-*H. pylori-* associated**	
	**Superficial chronic gastritis**	**Active chronic gastritis**	**Follicular chronic gastritis**	**Reactive gastritis**	***P-*value**
	*n*=75	*n*=37	*n*=35	*n*=17	
*H. pylori*					
Positive	14 (18.7)	9 (24.3)	21 (60)	6 (35.3)	<0.001*
Negative	61 (81.3)	28 (75.7)	14 (40)	11 (64.7)	
*vacA* genotypes					
*s2m2*	1 (7.1)	3 (33.3)	4 (19.1)	1 (16.7)	0.396*
*s1m2*	–	–	1 (4.8)	–	
*s1m1*	13 (92.9)	5 (55.6)	16 (76.1)	5 (83.3)	
*s1m1/s1m2*	–	1 (11.1)	–	–	
*cagA*					
Positive	12 (85.7)	5 (55.6)	16 (76.2)	4 (66.7)	0.383*
Negative	2 (14.3)	4 (44.4)	5 (23.8)	2 (33.3)	
*babA2*					
Positive	5 (35.7)	2 (22.2)	7 (33.3)	2 (33.3)	0.942*
Negative	9 (64.3)	7 (77.8)	14 (66.7)	4 (66.7)	

*Fisher's exact test.

### *cag*PAI negative clinical isolates

To corroborate the absence of *cag*PAI in the 13 *H*. *pylori cagA*^-^ strains, DNA of the isolates was subjected to the conventional PCR empty site assay. In 100 % (13/13) of *cagA*^-^ isolates, a 360 bp fragment was amplified (Fig. 1b), corroborating that 13 of the 50 patients (26 %) with chronic gastritis harboured *H. pylori* strains lacking *cag*PAI.

### Polymorphisms of *cagA* in EPIYA sequences

**Fig. 2.**
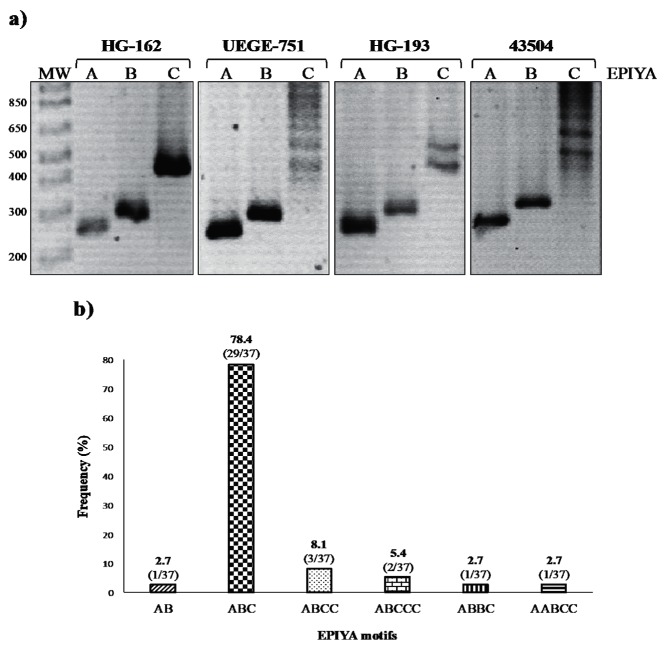
Characterization of EPIYA motifs. (a) Representative gel electrophoresis of PCR products of EPIYA motifs of strains: HG-162 (EPIYA-ABC), UEGE-751 (EPIYA-ABCCC) and HG-193 (EPIYA-ABCC). Strain ATCC43504 (EPIYA-ABCCC) was used as a positive control. Lanes: MW, 100 bp molecular weight marker; A, EPIYA-A motif (~256 bp); B, EPIYA-B motif (~306 bp); and C, EPIYA-C motif (first, 501 pb; second, ~650 pb; and third, >650 bp). (b) Frequency of CagA EPIYA motifs of *H. pylori* strains from patients with chronic gastritis.

**Fig. 3.**
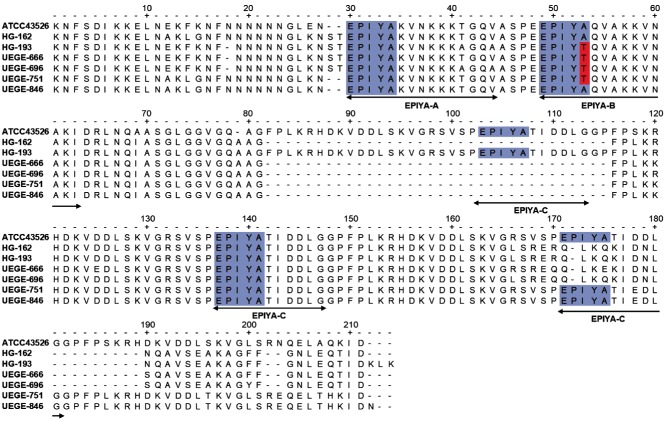
Alignment of the amino acid sequence of the C-terminal region of CagA from sequenced strains UEGE846 (EPIYA-ABCCC by PCR), UEGE751 (EPIYA-ABCCC by PCR), HG162 (EPIYA-ABC), HG193 (EPIYA-ABCC), UEGE666 (EPIYA-ABC) and UEGE696 (EPIYA-ABC). Sequence of strain ATCC43526 (EPIYA-ABCCC) was used as a reference. EPIYA sequences are highlighted in blue. Red boxes indicate alanine (A) to threonine (T) changes in EPIYA sequence. Arrows indicate EPIYA motifs.

To determine the type of EPIYA motifs of *H. pylori cagA*^+^ isolates, conventional PCR was performed using primers listed in Table 1. Six electrophoretic patterns were observed in the amplified fragments, corresponding to the combinations of EPIYA motifs: AB, ABC, ABCC, ABCCC, ABBC and AABCC (Fig. 2a). The number of EPIYA motifs varied among the 37 isolates, 2.7 % (1/37) contained two motifs (AB), 78.4 % (29/37) contained three (ABC), 10.8 % (4/37) contained four (ABCC and ABBC), and 8.1 % (3/37) contained five (ABCCC and AABCC) (Fig. 2b). All the isolates with three motifs had the combination ABC. Four strains (10.8 %) carried two EPIYA-C motifs, three had the ABCC combination, and the fourth had two A sequences (Table 5). In two male patients, *H. pylori cagA*^+^ with EPIYA-ABCCC motifs was isolated. With respect to the gastric pathology, two isolates with EPIYA-ABCC motif were obtained from patients with follicular chronic gastritis, and one from superficial chronic gastritis. One strain with the EPIYA-ABCCC motif was isolated from a patient with reactive gastritis, and one from a patient with follicular chronic gastritis. The only strain with EPIYA-AABCC was isolated from a patient with superficial chronic gastritis. As expected, we found no strains with the EPIYA-D motif. These results were confirmed by sequencing the ~650 to ~850 bp fragment of the 3′ variable region of *cagA* of six randomly selected strains. The chosen strains contained EPIYA motifs -ABC (strains UEGE666, UEGE696 and HG162), -ABCC (strain HG193) and -ABCCC (strains UEGE846 and UEGE751), as well as the *vacA* variant *s1m1*. Unexpectedly, only two EPIYA-C were detected in sequences of strains UEGE846 and UEGE751. Besides corroborating that our isolates belonged to the Western type, sequencing results showed that 66.7 % (4/6) of the strains carried the variant EPIYT-B motif (Fig. 3). This variant was found in two strains containing the -ABC motif (UEGE666, UEGE696), one with the -ABCC motif (HG193) and one with the -ABCCC motif (UEGE751) (Table 4). These four strains had the genotype *vacA s1m1*/*babA2*^+^.

**Table 5.** Age and gender of 37 patients with chronic gastritis and characteristics of the *H. pylori* strains isolated from them

**Strain**	**Age**	**Gender**	***vacA***	***babA2***	***cagA***	**EPIYA motifs**
HG02	63	M	*s1/m1*	+	+	ABC
HG43	54	M	*s1/m1*	−	+	ABC
HG44	43	F	*s1/m1*	+	+	ABBC
HG51	19	F	*s1/m1*	−	+	ABC
HG65	22	F	*s1/m1*	−	+	ABC
HG66	27	F	*s1/m1*	−	+	ABC
HG70	35	F	*s1/m1*	−	+	ABC
HG150	57	M	*s1/m1*	−	+	ABC
HG155	89	M	*s1/m1*	+	+	ABC
HG162	19	F	*s1/m1*	+	+	ABC
HG171	54	M	*s1/m1*	+	+	AABCC
HG177	37	F	*s1/m1*	−	+	ABC
HG179	65	F	*s1/m1, s1/m2*	−	+	ABC
HG189	30	F	*s1/m1*	−	+	AB
HG190	45	M	*s1/m1*	−	+	ABC
HG193	31	F	*s1/m1*	+	+	AB*CC
HG199	44	F	*s1/m1*	+	+	ABC
HG200	42	F	*s1/m1*	+	+	ABC
HG210	62	M	*s1/m1*	−	+	ABCC
HG211	43	M	*s1/m1*	−	+	ABC
HG224	50	F	*s1/m1*	−	+	ABC
UEGE563	75	F	*s1/m1*	−	+	ABC
UEGE640	32	M	*s1/m2*	−	+	ABC
UEGE652	71	F	*s1/m1*	−	+	ABC
UEGE666	52	M	*s1/m1*	+	+	AB*C
UEGE696	42	M	*s1/m1*	+	+	AB*C
UEGE740	41	F	*s1/m1*	−	+	ABC
UEGE748	57	M	*s1/m1*	−	+	ABC
UEGE751	45	M	*s1/m1*	+	+	AB*CCC
UEGE752	3	M	*s1/m1*	+	+	ABC
UEGE753	84	F	*s1/m1*	−	+	ABC
UEGE826	65	F	*s1/m1*	−	+	ABC
UEGE843	58	M	*s2/m2*	+	+	ABCC
UEGE845	27	M	*s1/m1*	+	+	ABC
UEGE846	27	M	*s1/m1*	−	+	ABCCC
UEGE847	65	F	*s1/m1*	+	+	ABC
UEGE852	62	M	*s1/m1*	−	+	ABC

F, female; M, male; B*, EPIYT-B motif.

## Discussion

*H. pylori* infection is associated with the development of gastric pathologies, and the frequency of infection varies among regions. In the present study, *H. pylori* was isolated with a frequency of 30.5 % (50/164), which is lower than that reported by Paniagua *et al*. in 2009 (60.1 %) in Mexican patients with chronic gastritis [29], but higher than those reported by Chihu *et al*. in 2005 (16.7 %) [30] in patients with chronic active gastritis, and Lopez-Vidal *et al*. in 2008 (26 %) [31] in patients with non-cancerous gastric diseases. These differences show the variability of *H. pylori* distribution in different regions of Mexico.

The frequency of *H. pylori* was significantly different between the types of chronic gastritis (*P*<0.001). The prevalence of infection in cases of reactive gastritis (35.3 %) was higher than in cases of superficial chronic gastritis (18.7 %) and active chronic gastritis (24.3 %), but lower than in cases of follicular chronic gastritis (60 %). The frequency of *H. pylori* in reactive gastritis is similar to that reported for Chilean patients (33 %) [32], but it contrasts with that found in Colombian patients (18.5 %) [33]. These differences can be explained by the geographic origin, the genetic background and the socio-demographic characteristics of the populations. In this research we analysed patients that attended the Gastroenterology Service at the General Hospital ‘Dr Raymundo Abarca Alarcón’ and the Specialized Unit in Gastroenterology Endoscopy; the first one serves people with very low socio-economic level and no access to social security services, while the latter attends people with better socio-economic conditions since it is a particular clinic.

*H. pylori* negative samples were more prevalent in all types of gastritis except follicular chronic gastritis. Actually, the majority of the isolates (42 %, 21/50) were obtained from patients with this type of gastritis, while only 12 % (6/50) were from patients with reactive gastritis, a type of non- *H. pylori-*associated gastritis [23]. This result suggests a mixed etiology in these patients, which have a higher risk of developing a gastric ulcer due to the synergic effect of the etiological agents [33, 34]. On the other hand, the differences in the frequency of *H. pylori* isolates by type of gastritis may be due to the distribution of the bacteria on the gastric mucosa, to the site where the biopsy was taken, and to the number of viable bacteria on the tissue.

Evidence indicates that the isoform *s1m1* of *vacA*, the presence of the *babA2* gene, and the type and number of the EPIYA motifs that characterize the *cagA* variants, are involved in the type and magnitude of the histological damage of the mucosa, for example, the *vacA s1m1* genotype has been associated with intestinal metaplasia, severe inflammation and high risk of gastric cancer [35]. In this study, the most virulent *vacA* allele, *s1m1*, was the most frequent (80 %) in the isolated strains. This frequency is similar to those found for populations from other regions in Mexico [29, 36–38], and reaffirms the frequencies previously determined in gastric biopsies from patients from Guerrero, Mexico [25, 39, 40].

The presence of *babA2*, which encodes the *H. pylori* active protein BabA, has been associated with peptic ulcer disease and gastric cancer in Western countries [41]. In this work, the frequency of *babA2*^+^ was 32 %, which is higher than that reported in 2009 for Mexican patients with chronic gastritis [29], but lower than that reported from isolated strains from pediatric patients between 10 months and 17 years old from Mexico City [42]. We are unable to rule out the possibility that the frequency of *babA2*^+^ found in this work was not influenced by the existence of *babA2* allelic variants not detected with the primers used. Of the *babA2*^+^ strains, 93.7 % (15/16) carried the *vacA s1m1* allele. Our results showed that the *vacA s1m1*/*cagA*^+^/*babA2*^+^ genotype was found in 28 % of the isolated strains. This frequency is lower than that found in Mexican pediatric patients (47.5 %) [42]. VacA and BabA proteins produced by *vacA s1m1*/*babA2*^+^ strains have a synergistic effect on *H. pylori* virulence, increasing the risk of a severe gastric disease [35].

*cagA*^+^ strains were found in 74 % of the isolated strains, a frequency similar to that found by Reyes-Leon *et al*. in 2008 (78.6 %) in strains from pediatric patients with chronic abdominal pain and adults with non-ulcerous dyspepsia or peptic ulcer [43], but higher than that found by Paniagua *et al*. in 2009 (52.4 %) by multiplex PCR [29]. In pediatric patients from Mexico City the frequency of *cagA*^+^ strains has reached 90.6 % [42]. In strains from Colombian patients with diverse gastric pathologies, the frequency of *cagA*^+^ strains is up to 83.8 % [44]. The presence of *cagA* was found more frequently along with the *vacA s1m1* variant (70 %), which is consistent with previous reports [45–47].

The most frequent EPIYA motif found in our isolated strains was -ABC (78.4 %), which is in agreement with previous reports from Mexican patients with gastric disease by Beltran-Anaya *et al*. and Rizzato *et al*. [28, 48]. This motif was found in a lower proportion (50 %) in Mexican pediatric patients [42]. Only two of the 37 isolated strains (5.4 %) carried the EPIYA-ABCCC motif, a proportion that is lower than that reported by Mendoza-Elizalde *et al*. in 2015 (18.75) [42], but higher than that found by Rizzato *et al*. in 2012 (3.7 %) in strains from Mexican and Venezuelan patients with chronic gastritis [48]. In 18.9 % of the isolated strains the number of EPIYA motifs was ≥4, and of these, 16.2 % had two or three EPIYA-C motifs. In the two isolations with three EPIYA-C by PCR the third C motif could not found by sequencing. The reason for this is not clear. Four out of six sequenced strains had a modified EPIYT-B motif, which is the most frequent EPIYA-B alternative in Western strains [49]. It has been shown that the EPIYT-B motif is associated with duodenal ulcers, and with induction of lower levels of cellular elongation and IL-8 secretion [43, 49]. This modified motif has been found before in Mexico in two different regions, being more prevalent in the state of Guerrero [28, 42].

It is probable that in this geographic region of Mexico, strains with the modified EPIYT-B motif are the most frequent, although this should be addressed in more detail.

Patients infected with *H. pylori cagA*^+^ strains that carry more than one EPIYA-C motif have a higher risk of developing atrophic gastritis and gastric carcinoma, since CagA possesses more C-terminal phosphorylation sites, a characteristic associated with a higher carcinogenic potential [49]. However, it is possible that this effect could be attenuated by the EPIYT modification in the EPIYA-B motif, and that this CagA variant attenuates the pathogenic effect caused by the three or more EPIYA-C motifs in patients infected with *vacA s1m1*/*babA2*^+^ strains.

In conclusion, our results document an important diversity of Western variants of *cagA* in *H. pylori* strains isolated from patients with chronic gastritis. The prevalence of *H. pylori* is significantly different between the different types of chronic gastritis, and in these, the genotypes *vacA s1m1*/*cagA*^+^ are the most prevalent. The 3′ variable region of *cagA*, and thus the CagA protein of *H. pylori* strains from the south of Mexico is heterogeneous in the number and type of EPIYA motifs. In these *H. pylori* isolates, the *vacA s1m1* genotype along with *cagA* variants encoding EPIYA-ABC patterns were predominant, and a significant proportion of these were *babA2*^+^. *H. pylori* strains containing the EPIYT-B motif in combination with one or more C motifs are common in patients with chronic gastritis.
